# The Methyltransferase Smyd1 Mediates LPS-Triggered Up-Regulation of IL-6 in Endothelial Cells

**DOI:** 10.3390/cells10123515

**Published:** 2021-12-13

**Authors:** Ahmed Shamloul, Gustav Steinemann, Kerrin Roos, Celine Huajia Liem, Jonathan Bernd, Thorsten Braun, Andreas Zakrzewicz, Janine Berkholz

**Affiliations:** 1 Shear Stress and Vascular Biology Research Group, Institute of Physiology, Charité-Universitätsmedizin, Corporate Member of Freie Universität Berlin, Humboldt-Universität zu Berlin, Charitéplatz 1, 10117 Berlin, Germany; ahmed.shamloul@charite.de (A.S.); gustav.steinemann@charite.de (G.S.); kerrin.roos@charite.de (K.R.); celine.liem@charite.de (C.H.L.); jonathan.bernd@charite.de (J.B.); andreas.zakrzewicz@charite.de (A.Z.); 2 Department of Obstetrics, Charité-Universitätsmedizin Berlin, Corporate Member of Freie Universität Berlin, Humboldt-Universität zu Berlin, Augustenburger Platz 1, 13353 Berlin, Germany; thorsten.braun@charite.de; 3 DZHK (German Centre for Cardiovascular Research), Partner Site Berlin, 10785 Berlin, Germany

**Keywords:** Smyd1, lipopolysaccharide, sepsis, IL-6, NF-κB, histone methylation, endothelial cells

## Abstract

The lysine methyltransferase Smyd1 with its characteristic catalytic SET-domain is highly enriched in the embryonic heart and skeletal muscles, participating in cardiomyogenesis, sarcomere assembly and chromatin remodeling. Recently, significant Smyd1 levels were discovered in endothelial cells (ECs) that responded to inflammatory cytokines. Based on these biochemical properties, we hypothesized that Smyd1 is involved in inflammation-triggered signaling in ECs and therefore, investigated its role within the LPS-induced signaling cascade. Human endothelial cells (HUVECs and EA.hy926 cells) responded to LPS stimulation with higher intrinsic Smyd1 expression. By transfection with expression vectors containing gene inserts encoding either intact Smyd1, a catalytically inactive Smyd1-mutant or Smyd1-specific siRNAs, we show that Smyd1 contributes to LPS-triggered expression and secretion of IL-6 in EA.hy926 cells. Further molecular analysis revealed this process to be based on two signaling pathways: Smyd1 increased the activity of NF-κB and promoted the trimethylation of lysine-4 of histone-3 (H3K4me3) within the IL-6 promoter, as shown by ChIP-RT-qPCR combined with IL-6-promoter-driven luciferase reporter gene assays. In summary, our experimental analysis revealed that LPS-binding to ECs leads to the up-regulation of Smyd1 expression to transduce the signal for IL-6 up-regulation via activation of the established NF-κB pathway as well as via epigenetic trimethylation of H3K4.

## 1. Introduction

Like most other lysine methyltransferases, the members of the Smyd gene family express a characteristic SET domain, which contains the active site required for protein methylation using *S*-adenosyl methionine (SAM) as a methyl group donor [1,2]. Remarkably, only in the case of the Smyd proteins is the catalytic SET domain split by a MYND domain [3]. The five proteins of the Smyd gene family were originally discovered in embryonic heart and skeletal muscles, where they were found to participate in cardiomyogenesis and sarcomere assembly [4]. However, it is now clear that their tissue distribution and their functional relevance is much more complex than initially reported, as Smyd proteins are involved in many physiological processes, such as chromatin remodeling, transcription, signal transduction and cell cycle control, due to multiple interactions with other molecules [3,4]. 

In principle, five different types of molecular mechanisms of the Smyd proteins are distinguishable. (1) By the joint action of the two spatially separated SET half domains, four of the five Smyd family members methylate lysines (K) in histones (H) at selected positions, such as H3K4, H3K36 or H4K5 [4,5,6]. The complexity of these histone modifications is increased by the fact that most of the lysines can be methylated once (me1), twice (me2) or even three times (me3). These posttranslational modifications of histones cause conformational changes of the nucleosome, which might directly alter the accessibility of the promoter sequences for the transcription apparatus [7]. Alternatively, according to the histone code [8], methylated histones might be recognized by factors that then indirectly trigger changes in the transcription frequency of the downstream target genes [9]. (2) The SET domain-related methyltransferase activity of Smyd proteins methylates other proteins than histones in order to influence their activity/function [10]. (3) Smyd proteins adhere directly to/interact with DNA promoter sequences for the steric inhibition of the transcription apparatus or increasing their methyltransferase activity [11,12]. (4) With their MYND-type zinc finger domain, Smyd proteins interact with other proteins, e.g., to recruit transcription co-repressors [13,14] or transcription factors [9]. (5) The C-terminal domain (CTD) of Smyd proteins inhibits their methyltransferase activity as an intrinsic autoinhibitory loop [15].

Among the members of the Smyd gene family, Smyd1 is the only protein identified so far that is expressed in endothelial cells (ECs) under non-pathological conditions [16,17]. ECs outline the inner surface of the blood vessels and, therefore, represent important barriers between the bloodstream and extravascular tissue. Smyd1 influences the migration activity and tube formation of ECs and thus might have an impact on angiogenesis [16]. Furthermore, the Smyd1 expression in ECs is influenced by the cytokines IFN-γ and TNF-α [17], suggesting that this member of the Smyd family is sensitive to inflammation. 

Inflammatory reactions in humans are frequently triggered by lipopolysaccharides (LPS). Physiologically, LPS are large molecules of the outer cell membrane (as part of the cell wall) of Gram-negative bacteria. However, when these bacteria are pathologically released into the circulation of a human, LPS may shed from the cell wall to be released in the circulatory system of the host, where they finally interact with specific receptors on the EC surface of all blood vessels [18]. As a result, molecular signaling cascades are initiated, which include the induction/suppression of numerous relevant downstream molecules in these ECs, e.g., adhesion molecules, such as E-selectin and ICAM1 [19,20], or proinflammatory cytokines, such as IL-1β and IL-6 [21,22]. Taken together, LPS act as endotoxins in hosts, evoking several pathophysiological reactions in the vascular system, e.g., the breakdown of the barrier function [23], apoptosis of ECs [24] and stimulation of coagulation cascades [25] with further progression of organ dysfunctions, which can ultimately lead to the development of fatal sepsis [26,27].

Given that the inflammatory-sensitive signaling molecule Smyd1 is expressed in ECs, which are reactive to LPS exposition, we hypothesized that Smyd1 is integrated into the LPS signaling cascade and is therefore involved in the regulation of IL-6 expression. To validate this hypothesis, we systematically examined the relationship between LPS, Smyd1 and IL-6 in human EC cultures. By molecular analysis of EA.hy926 cells transfected with Smyd1 vectors or Smyd1-specific siRNAs, we found that LPS induced Smyd1-dependent trimethylation and activation of the IL-6 promoter, suggesting an upstream effect of Smyd1 on IL-6 expression in ECs.

## 2. Materials and Methods

### 2.1. Cell Culture

EA.hy926 cells (Elabscience, Biotechnology Inc., Houston, TX, USA) were cultured in Dulbecco’s modified Eagle’s medium (Gibco, Carlsbad, CA, USA) containing 10% fetal bovine serum (growth medium). Human umbilical vein endothelial cells (HUVECs) were isolated as described [28] with approval of the Ethics Committee of the Charité—Universitätsmedizin Berlin, Germany (EA4/107/17), and cultured in Endothelial Cell Growth Medium (PromoCell, Heidelberg, Germany). Umbilical cords were obtained from healthy mothers with written informed consent in accordance with the Declaration of Helsinki. All cells were maintained at 37 °C in a humidified atmosphere of 5% CO_2_.

### 2.2. Cell Stimulation Experiments with LPS or IL-6

LPS stimulation experiments, EA.hy926 cells and HUVECs were incubated with different concentrations of LPS (in the range of 1 ng/mL to 10 µg/mL; Sigma Aldrich, St. Louis, MO, USA). LPS stock solutions (1 mg/mL) were dissolved in PBS, pH 7.4 containing 1% bovine serum albumin (BSA) as carrier. For the study of the effect of IL-6 on Smyd1 expression in EA.hy926 cells, recombinant human IL-6 (PeproTech, Hamburg, Germany) diluted in PBS, pH 7.4 containing 1% BSA was used in a final concentration of 10 ng/mL. Both LPS and IL-6 were added to the cells with gentle mixing, to be then incubated for 3 h or 24 h, respectively. The control cells were treated with PBS/BSA.

### 2.3. Treatment of Cells with PDTC or Bay 11-7082

Pyrrolidine dithiocarbamate (PDTC, final concentration: 10 μM; Roth, Karlsruhe, Germany), and Bay 11-7082 (final concentration: 10 μM; Callbiochem, San Diego, CA, USA), both dissolved in dimethyl sulfoxide (DMSO), were added to the medium. DMSO alone served as a negative control. 

### 2.4. Transfection with Human Expression Vectors 

The transfection of EA.hy926 cells with expression plasmids was performed as previously described [29]. For transient transfection of the full-length, active Smyd1 expression plasmid into EA.hy926 cells, the pCMV2-Smyd1-flag (Sino Biological, Beijing, China) plasmid and of the methyltransferase-silent Smyd1 variant, the pBK-CMV-Smyd1_HMTase mutant_ (↑Smyd1 SET Mut) plasmid in which the Smyd1 gene insert contained a point mutation within the SET domain were used in combination with the TurboFect reagent (Thermo Fisher Scientific, Waltham, MA, USA) according to the manufacturer’s instructions. Briefly, EA.hy926 cells were transfected with 1 µg DNA diluted in 100 µL Opti-MEM (Gibco) for 24 h when cells reached ~70% confluence. Transfection efficiency was tested by RT-qPCR and by immunoblot analysis. pBK-CMV-Smyd1_HMTase mutant_ was a kind gift from Haley O. Tucker, Department of Molecular Biosciences, University of Texas at Austin, U.S.A. The term “control cells” refers to cells transfected with the corresponding vector lacking a specific gene insert.

### 2.5. Transfection with Specific siRNAs

The transfection of EA.hy.926 cells was performed, using a mixture of four unrelated siRNA species (25 nM final concentration) directed against the Smyd1 nucleotide sequence (Dharmacon, Lafayette, CO, USA; Cat.No: L-022738-01-0005). A non-gene-specific “scrambled” siRNA was used as a negative control. EA.hy926 cells were transfected using the transfection reagent Interferin (Polyplus Transfection, Illkirch-Graffenstaden, France), according to the manufacturer’s instructions. Knockdown of the target mRNA was monitored 24 h after transfection with siRNAs by RT-qPCR and immunoblotting. 

### 2.6. RNA Isolation, Reverse Transcription and Real Time qPCR

RNA was extracted from EA.hy926 and HUVECs using the GeneMATRIX Universal RNA Purification kit from EURx according to the manufacturer’s instructions. Semi-quantitative RT-qPCR analysis was carried out using the QuantStudio 5 system (Applied Biosystems, Foster City, CA, USA). Amounts of specific cDNAs were determined using the GoTaq qPCR Master Mix (Promega, Madison, WI, USA). Primer sequences, product sizes and annealing temperatures are listed in Table 1. In each experiment, melting curve analysis was performed to verify that a single transcript was produced. RT-qPCR relative mRNA levels were calculated using the comparative CT (2^−ΔΔC^_T_) method, with GAPDH as a reference. Non-RT- and non-template controls were run for all reactions. 

### 2.7. Immunoblotting

Cells were homogenized in RIPA buffer (Santa Cruz, Biotechnology, Dallas, TX, USA) containing standard protease inhibitors (Sigma-Aldrich) and 20 mM N-ethylmaleimide (Sigma-Aldrich) for 15 min at 4 °C. Cell lysates were collected after centrifugation to be subjected to protein concentration determination using the BCA Protein Assay Kit (Thermo Fisher Scientific). Total protein extracts (20 μg) were resolved by SDS-PAGE gel electrophoresis and transferred to nitrocellulose membranes (GE Healthcare, Chicago, IL, USA) for immunoblotting. Cytoplasmic and nuclear extracts were prepared using the NE-PER Nuclear and Cytoplasmic Extraction Kit (Thermo Fisher Scientific) according to the manufacturer’s instructions. Primary antibodies were diluted as follows: anti-Smyd1 (1:1000, Thermo Fisher Scientific, Cat.No: PA5-31482; rabbit), anti-IL-6 (1:500, Santa Cruz Biotechnology, Cat.No: sc-28343; mouse), anti- NF-κB p65 (1:1000, Cell Signaling Technology, Danvers, MA, USA; Cat.No: 8242S; rabbit), anti-IκBα (1:1000, Cell Signaling Technology, Cat.No: 4814S; mouse), anti-GAPDH (1:10,000, Proteintech, Rosemont, IL, USA; Cat.No: HRP-60004; mouse), anti-Emerin (1:2000, Abcam, Cambridge, UK; Cat.No: ab153718; rabbit). A peroxidase-conjugated secondary antibody (1:1000, Santa Cruz) was used to detect antibodies bound to the blot matrix and visualized using a chemiluminescence kit (Bio-Rad, Hercules, CA, USA). 

### 2.8. Immunofluorescence Analysis

Cellular localization of Smyd1 and NF-κB -p65 was assessed on fixed EA.hy926 cells by immunocytochemistry combined with confocal laser scanning microscopy. Cells were fixed with 4% paraformaldehyde in PBS, pH 7.4 for 15 min at room temperature and permeabilized by PBS, pH 7.4, containing 0.5% Triton X-100. Nuclei were stained with Draq5 (1:1000, Thermo Fisher Scientific). After blocking with 10% FCS in PBS, pH 7.4, fixed cells were incubated overnight at 4 °C with an anti-Smyd1 (1:100, Thermo Fisher Scientific, Cat.No: PA5-31482; rabbit), an anti-NF-κB-p65 antibody (1:100, Cell Signaling, Cat.No: 8242S; rabbit) and/or an anti-alpha Tubulin-1 (1:100, D-11, Santa Cruz, Cat.No: sc-32293; mouse) antibody in blocking buffer. Alexa Fluor-conjugated secondary antibodies (Life Technologies, Carlsbad, CA, USA) were diluted in blocking buffer containing 5 µM Draq5. Cells were incubated with the respective secondary antibody mix for 1 h at room temperature, washed 3 times with PBS, pH 7.4, and covered with fluorescence mounting medium (1:2000, Agilent Technologies, Santa Clara, CA, USA). Isotype-matched, non-binding primary IgG antibodies of the same species in the same concentration as the specific primary antibodies served as negative controls. Stained cells were then assessed with a confocal laser microscope (Leica DMI 6000, Wetzlar, Germany) equipped with 20× and 63× oil immersion lens, a single-photon argon laser, a solid-state laser and a helium-neon laser. The Leica LAS AF Lite software was used to process the digital images and analyze the mean fluorescence intensity (MFI). For MFI quantification, all images were analyzed using the same optical settings. The MFI was measured in 20 regions of interest (ROIs) per experiment belonging to either the nucleus or the cytoplasmic area. A non-fluorescent region of the same image was analyzed as background MFI and then subtracted from each of the MFI in the specific ROIs.

### 2.9. ELISA Analysis

IL-6 and IL-8 protein concentrations were determined in cell culture supernatants using commercial human ELISA kits (Invitrogen, Waltham, MA, USA) according to the manufacturer’s guidelines. Samples were analyzed in triplicate.

### 2.10. Luciferase Assay Reporter Gene Assay

EA.hy926 cells were transiently transfected with pBABE lucIL6 reporter or NF-κB luciferase reporter vector (BPS Bioscience, San Diego, MA, USA) and partially transfected in parallel with the wild-type expression plasmid pCMV2-Smyd1-flag (Sino Biological) or the pBK-CMV-Smyd1_HMTase mutant_ plasmid using TurboFect transfection reagent (Thermo Fisher Scientific) according to the manufacturer’s instructions. pBABE lucIL6 was a kind gift from Sheila Stewart (Addgene plasmid # 52884) [30]. The vector contains the human IL-6 promoter region upstream of the luciferase-encoding sequence of plasmid pBABE hygro. NF-κB luciferase reporter vector contains a firefly luciferase gene under the control of a multimerized NF-κB responsive element located upstream of a minimal promoter. Stimulation with LPS (1 µg/mL) served as the positive control. After 24 h, the cells were harvested in lysis buffer and assayed for firefly activity as described by the manufacturer (Promega). The amount of luciferase activity in each sample was quantified in a Varioskan Flash Multimode Reader (Thermo Fisher Scientific).

### 2.11. Flow Cytometry

After centrifugation (1000 rpm for 5 min at 4 °C), pelleted EA.h926 cells were resuspended in PBS and then fixed in methanol for 20 min at −20 °C. Subsequently, cells were blocked in 1% FCS in PBS for 1h and then incubated with the primary non-conjugated antibodies (anti-Smyd1, Santa Cruz; anti-NF-κB-p65, Cell Signaling) or isotype-matched monoclonal IgG control antibodies, all diluted in 1% FCS in PBS at 4 °C for 2 h. Subsequently, the cells were washed twice with 1% FCS in PBS and stained with the secondary antibodies conjugated to Alexa Flour 488 or 594, diluted in PBS (1:1000) with 1% FCS at 4 °C for 1 h. The flow cytometric analysis was carried out after two additional washing steps with a FACS Calibur (BD Biosciences, Franklin Lakes, NJ, USA) using CellQuest software. 

### 2.12. ChIP RT-qPCR Assays

ChIP-RT-qPCR assays with transfected or LPS-stimulated EA.hy926 cells were performed using the EpiQuik Chromatin Immunoprecipitation Kit (Epigentek, Farmingdale, NY, USA) following the protocol supplied by the manufacturer. For the ChIP assays, 1 × 10^6^ cells were fixed in 1% formaldehyde for 10 min. Lysed samples were sonicated to fragment the DNA. The DNA-bound proteins were immunoprecipitated with antibodies directed against H3K4me1, H3K4me2 and H3K4me3 (Epigentek) or Smyd1 (Thermo Fisher Scientific). The collected DNA and input samples were analyzed for associated DNA fragments using quantitative RT-qPCR. Previous work by other groups [31,32,33] showed that the transcription frequency of IL-6 is related to the state of different regions present in the IL-6 promoter active in different cell types, in particular to that of three distinct CpG islands: a cluster at approximately −1500/−1300 bp (8 CpG sites), a cluster at approximately −670/−400 bp (7 CpG sites) and a cluster at approximately 100/150 bp relative to the transcription start (10 CpG sites). It was shown, for example, that the qualitative and quantitative modification patterns of histone H3 on each of these three CpG islands had a significant influence on the IL-6 transcription rates in synovial fibroblasts [33]. Therefore, ChIP RT-qPCR analysis with primer pairs that cover DNA sequences in the vicinity of these three CpG islands, primer pair 1 at −1522 bp to −1291 bp, primer pair 2 at −691 bp to −399 bp and primer pair 3 at 96 bp to 293 bp of the IL-6 gene, were carried out. The primer sequences, PCR product sizes and position within the IL-6 gene used in ChIP-RT-qPCR are listed in Table 2. 

### 2.13. Statistical Analysis

Data were analyzed with GraphPad Prism software 8.0 (Graph-Pad Software, San Diego, CA, USA) and presented as mean ± SD (*n* ≥ 3). All data sets were tested by Shapiro–Wilk for their normality of distribution prior to statistical analysis and the Brown–Forsythe test was performed to test the equality of variance. Comparisons between the two groups were performed by Student *t*-test (2-tailed unpaired), between >2 groups by one-way ANOVA followed by the Dunnett test or by two-way ANOVA followed by the Bonferroni post hoc test. 

## 3. Results

### 3.1. LPS Stimulation of Endothelial Cells Leads to Higher Smyd1 Expression 

**Figure 1:**
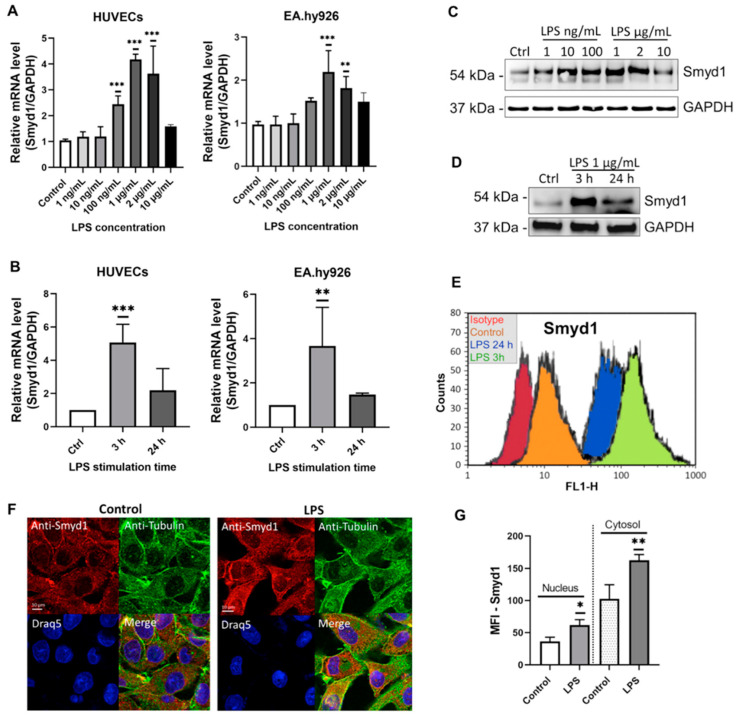
LPS stimulation increases Smyd1 expression in endothelial cells. (**A**) RT-qPCR for the quantification of Smyd1 mRNA levels in HUVECs and EA.hy926 cells that were stimulated with different concentrations of LPS ranging from 1 ng/mL to 10 µg/mL for 3 h. Expression values relative to control (no LPS supplement). *n* = 3, ** *p* < 0.01, *** *p* < 0.001 using one-way ANOVA. (**B**) RT-qPCR for the quantification of Smyd1 mRNA levels in HUVECs and EA.hy926 cells that were stimulated with 1 µg/mL LPS for 3 h or 24 h. Expression values relative to control (Ctrl, no LPS supplement). *n* = 3, ** *p* < 0.01, *** *p* < 0.001 using one-way ANOVA. (**C**) Immunoblotting for the determination of Smyd1 protein levels in total lysates of EA.hy926 cells incubated with different concentrations of LPS ranging from 1 ng/mL to 10 µg/mL for 3 h in comparison to no LPS supplement (Ctrl). Representative immunoblot of *n* = 3. (**D**) Immunoblotting for the determination of Smyd1 protein levels in total lysates of EA.hy926 cells after incubation with 1 µg/mL LPS for 3 h or 24 h in comparison to no LPS supplement (Ctrl). Representative immunoblot of *n* = 3. (**E**) Representative flow cytometry histograms of EA.hy926 cells stimulated without LPS or with 1 µg/mL LPS for 3 h or 24 h and incubated with a monoclonal antibody recognizing Smyd1 and, subsequently, fluorescence-labeled secondary antibodies. An isotype-matched monoclonal IgG was used as control. *n* = 3. (**F**) Immunocytochemistry with anti-Smyd1 (red) and anti-Tubulin (green) antibodies on EA.hy926 cells incubated for 3 h with vehicle only (Control) or LPS (1 µg/mL). Draq5 staining for labeling of cell nuclei (blue). Representative images of *n* = 3 experiments. (**G**), Mean fluorescence intensity (MFI) of Smyd1 immunoreactivity, as shown in F, was densitometrically determined in the nucleus (left two columns) and the cytosol (right two columns). *n* = 3 independent experiments each with 20 cells evaluated. * *p* < 0.05, ** *p* < 0.01 using Student *t*-test. All graphs reported as mean ± SD.

To investigate whether LPS has an effect on Smyd1 expression in endothelial cells (ECs), HUVECs and EA.hy926 cells were incubated without LPS (control) or with different concentrations of LPS (in the range of 1 ng/mL to 10 µg/mL) for 3 h. This LPS treatment led to a dose-dependent increase in Smyd1 expression at the mRNA level (*p* < 0.01), which peaked at 1 µg/mL (Figure 1A; HUVECs: +321%; EA.hy926 cells: +123%). Higher Smyd1 mRNA concentrations (*p <* 0.01) were expressed after 3 h (HUVECs: +397%; EA.hy926 cells: +290%) than 24 h in both HUVECs and EA.hy926 cells during stimulation with 1 μg/mL LPS (Figure 1B). Corresponding results (peak in Smyd1 expression at 1 µg/mL (Figure 1C and Figure S1A)) after 3 h of LPS stimulation (Figure 1D and Figure S1B) were found at the protein level when EA.hy926 cell lysates were subjected to quantitative immunoblotting. Accordingly, flow cytometry analysis demonstrated higher intracellular Smyd1 levels in EA.hy926 cells after 3 h and 24 h of LPS stimulation than in non-stimulated cells (Figure 1E). Strong anti-Smyd1 immunoreactivity was observed in both the nuclear and cytoplasmic compartments by immunocytochemistry when EA.hy926 cells remained non-stimulated or were stimulated with LPS for 3 h (Figure 1F). However, the immunocytochemical signal was higher in the nucleus and the cytoplasmic compartment of LPS-stimulated compared to non-stimulated EA.hy926 cells (Figure 1F,G).

### 3.2. Smyd1 Increases IL-6 Levels in Endothelial Cells

**Figure 2:**
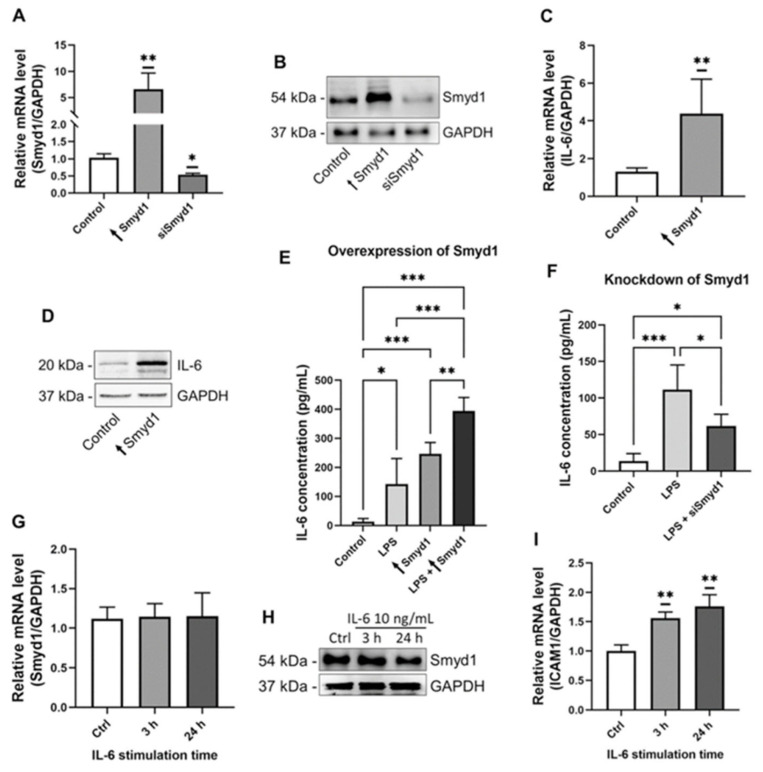
Smyd1 increases IL-6 expression and secretion in endothelial cells. (**A**,**B**) RT-qPCR to quantify Smyd1 mRNA levels (**A**) and immunoblotting for the determination of Smyd1 protein levels (**B**) in pCMV2-Smyd1-flag vector (↑Smyd1) or Smyd1-specific siRNA transfected EA.hy926 cells. Expression values relative to control (transfection with vector lacking gene insert). *n* = 3, * *p* < 0.05, ** *p* < 0.01 using one-way ANOVA. Representative immunoblot of *n* = 3. (**C**,**D**) RT-qPCR for the quantification of IL-6 mRNA levels (**C**) and immunoblotting for the determination of IL-6 protein levels (**D**) in total lysates of EA.hy926 cells, which were transfected with either a pCMV2-Smyd1-flag vector (↑Smyd1) or a vector without specific gene insert (Control). Expression values relative to control. *n* = 3, ** *p* < 0.01 using Student *t*-test. Representative immunoblot of *n* = 3. (**E**) ELISA for the quantification of IL-6 protein concentrations in supernatants of EA.hy926 cells transfected with a pCMV2-Smyd1-flag vector (↑Smyd1) or a vector lacking a specific gene insert (Control) without or with LPS (1 µg/mL) stimulation for 24 h. *n* = 7, * *p* < 0.05, ** *p* < 0.01, *** *p* < 0.001 using two-way ANOVA. (**F**) ELISA for the quantification of IL-6 protein concentrations in supernatants of EA.hy926 cells simultaneously transfected with Smyd1-specific siRNA and stimulated with LPS (1 µg/mL) for 24 h or scrambled siRNA (Control). *n* = 5, * *p* < 0.05, *** *p* < 0.001 using two-way ANOVA. (**G**,**H**) RT-qPCR to quantify mRNA levels (**G**) and immunoblotting for the determination of protein levels (**H**) of Smyd1 in EA.hy926 cells that were stimulated with 10 ng/mL IL-6 for 3 h or 24 h. Expression values relative to control (Ctrl; no IL-6 stimulation). *n* = 3, * *p* > 0.05 using one-way ANOVA and representative immunoblot of *n* = 3. (**I**) RT-qPCR to quantify ICAM1 mRNA levels in EA.hy926 cells that were stimulated with 10 ng/mL IL-6 for 3 h or 24 h. Expression values relative to control (Ctrl; no IL-6 stimulation). *n* = 3, ** *p* < 0.01 using one-way ANOVA. All graphs reported as mean ± SD.

Because LPS is an established trigger of IL-6 production and released in many cells, we next evaluated whether Smyd1 influences the expression of IL-6 in ECs. Therefore, the IL-6 expression levels were determined in EA.hy926 cells that were transfected with either a Smyd1 gene-containing vector or a vector lacking a specific gene insert (Figure 2A,B and Figure S1C). lL-6 expression was higher (*p <* 0.001) in the Smyd1-overexpressing cells than the control cells at both the mRNA (+344%; Figure 2C) and protein (+47%; Figure 2D and Figure S1D) level. 

As shown in Figure 2E,F, ELISA was carried out to quantify IL-6 concentrations in supernatants of EA.hy926 cells that were collected 24 h after transfection with a vector containing either the intact Smyd1 gene or without the specific gene insert (control) or Smyd1-specific siRNA or scrambled siRNA (control), each with simultaneous LPS stimulation. Compared to the supernatants of the control cells, IL-6 concentrations were higher (*p <* 0.05) in the supernatants of EA.hy926 cells either stimulated with LPS (+910%) or overexpressing Smyd1 (+1790%). The highest IL-6 levels were found in the supernatants of the EA.hy926 cells that were simultaneously LPS-stimulated and Smyd1-transfected (*p* < 0.001; +2990%; Figure 2E). In contrast, LPS stimulation led to lower IL-6 secretion (*p <* 0.05; −45%; Figure 2F) if the EAhy926 cells were treated with Smyd1-siRNA compared to experimentally non-manipulated cells stimulated with LPS.

To uncover a possible feedback regulation of IL-6 on Smyd1 expression, we quantified the Smyd1 levels in EA.hy926 cells after 3 h and 24 h of IL-6 stimulation, respectively. As shown in Figure 2G,H, RT-qPCR and immunoblotting (Figure S1E) revealed that IL-6 did not influence (*p* ≥ 0.05) Smyd1 expression levels (3 h: +7%, 24 h: +13%). In contrast, the expression of the established downstream target ICAM1 was up-regulated (*p* < 0.01) in IL-6 stimulated EA.hy926 cells (3 h: +56%, 24 h: +73%; Figure 2I).

### 3.3. Smyd1 Induces IL-6 Expression via Activation of NF-κB 

**Figure 3:**
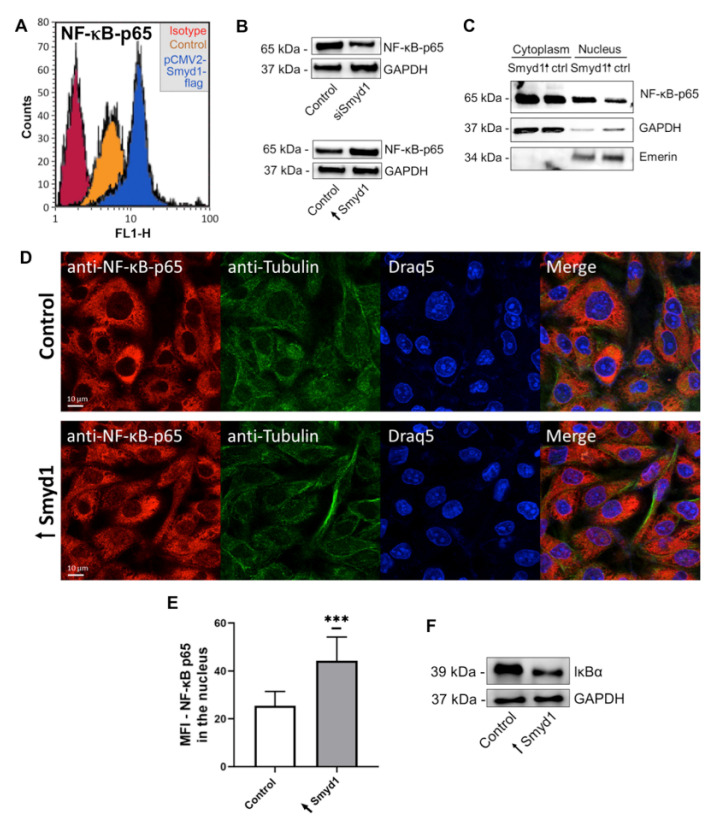
Smyd1 up-regulates and activates nuclear translocation of NF-κB in EA.hy926 cells. (**A**) Representative flow cytometry histograms of EA.hy926 cells transfected with a pCMV2-Smyd1-flag vector or a vector without gene insert (control) and incubated with a monoclonal antibody recognizing the p65 subunit of NF-κB and, subsequently, fluorescence-labeled secondary antibodies. An isotype-matched monoclonal IgG control antibody was used as control. (**B**) Immunoblotting for the determination of NF-κB-p65 levels in total lysates of EA.hy926 cells transfected either with a vector lacking a specific gene insert (Control) or a pCMV2-Smyd1-flag vector (↑Smyd1) and scrambled siRNA (Control) or Smyd1-specific siRNAs (siSmyd1). Representative immunoblots of *n* = 3. (**C**) Immunoblotting for the determination of NF-κB-p65 subunit levels in nuclear and cytoplasmic fractions of EA.hy926 cells (isolated by NE-PER extraction kit) that were transfected with a pCMV2-Smyd1-flag vector (↑Smyd1) or a vector without gene insert (Ctrl) for 24 h. Blot matrices were probed with antibodies against NF-κB p65, Emerin (nuclear marker) and GAPDH (cytoplasmic marker). Representative immunoblot of *n* = 3. (**D**) Immunocytochemistry of EA.hy926 cells with anti-NF-κB subunit (red) and anti-Tubulin (green) antibodies 24 h after transfection with a vector without gene insert (Control; upper panel) or a pCMV2-Smyd1-flag vector (↑Smyd1; lower panel). Draq5 (blue) was used to counterstain the cell nuclei. Representative images of *n* = 3. (**E**) Mean fluorescence intensity (MFI) of NF-κB p65 cytochemical immunoreactivity (as shown in **D**) was densitometrically determined in the nucleus. *n* = 3 independent experiments each with 20 cell nuclei evaluated. *** *p* < 0.001 using Student *t*-test. (**F**) Immunoblotting for the determination of IκBα protein levels in total lysates of EA.hy926 cells transfected with a pCMV2-Smyd1-flag vector (↑Smyd1) or a vector without specific gene insert (Control). Representative immunoblot of *n* = 3. All graphs reported as mean ± SD.

To test the hypothesis that Smyd1 increases IL-6 expression via NF-κB, the role of Smyd1 in the activation of NF-κB was examined. Flow cytometry analysis revealed that EA.hy926 cells transfected with a Smyd1-containing vector expressed higher levels of the NF-κB subunit RelA/p65 (NF-κB-p65) than control EA.hy926 cells that were transfected with a vector without gene insert (Figure 3A). 

The flow cytometry findings were validated by immunoblotting on total lysates from Smyd1-transfected EA.hy926 cells. As shown in Figure 3B and Figure S1F,G, the knockdown of Smyd1 led to lower NF-κB p65 immunoblotting levels, whereas the overexpression of Smyd1 resulted in higher NF-κB p65 levels compared to the NF-κB p65 levels in the control cells. Interestingly, NF-κB p65 was particularly enriched in the nuclear fraction (Figure 3C and Figure S1H) when lysates of EA.hy926 cells transfected with the Smyd1-gene-containing vector or the corresponding vector without gene insert were subjected to subcellular fractionation. Quantitative image analysis of immunocytochemical stainings also confirmed the NF-κB p65 nuclear translocation in Smyd1-overexpressing EA.hy926 cells (Figure 3D,E). Furthermore, Smyd1-overexpressing EA.hy926 cells showed lower IκBα protein levels than the control cells (Figure 3F and Figure S1I). 

### 3.4. Smyd1 Induces IL-6 Expression also Independently of NF-κB

**Figure 4:**
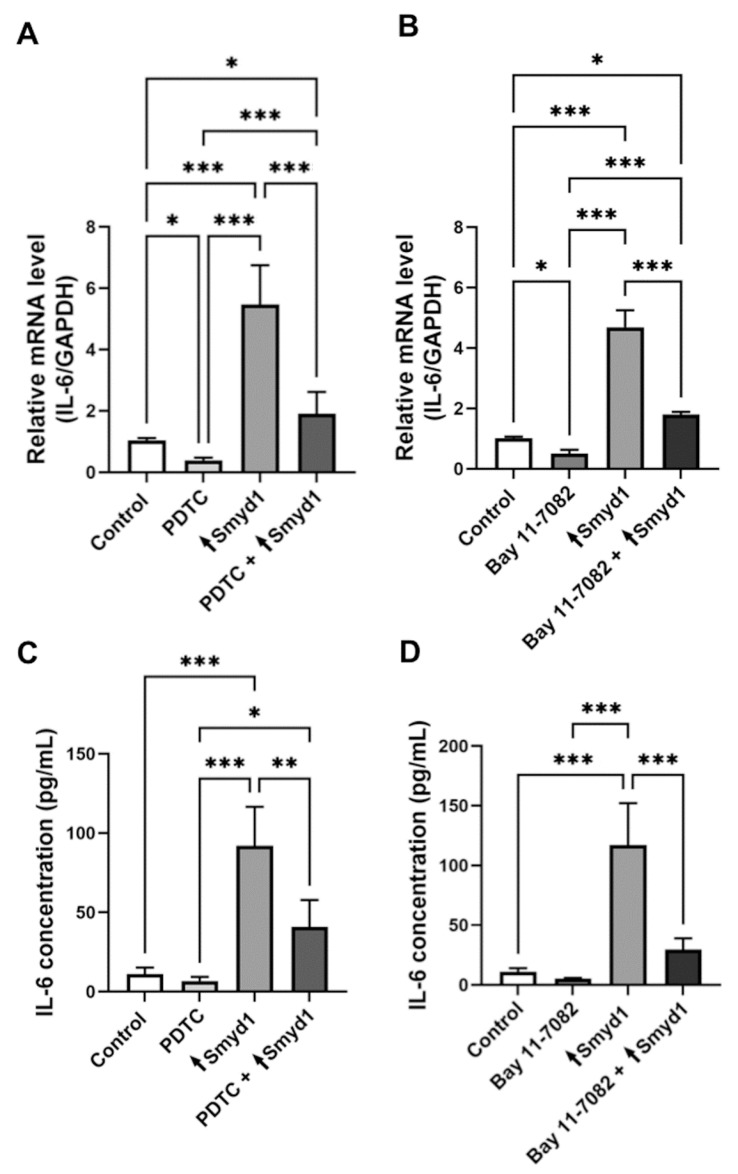
The inhibition of NF-κB activity does not completely abolish the increase of Smyd1 expression in EA.hy926 cells. (**A**,**B**) RT-qPCR for the quantification of IL-6 mRNA levels in EA.hy926 cell lysates transfected with a pCMV2-Smyd1-flag vector (↑Smyd1) and/or treated with the NF-κB inhibitors PDTC (10 μM) (**A**) or Bay 11-7082 (10 μM) (**B**). Expression values relative to the untreated control cell extracts *n* = 3, * *p* < 0.05, *** *p* < 0.001 using two-way ANOVA. (**C**,**D**) ELISA for the quantification of IL-6 concentrations in supernatants of EA.hy926 cells transfected with the pCMV2-Smyd1-flag vector (↑Smyd1) and/or treated with PDTC (10 μM) (**C**) or Bay 11-7082 (10 μM) (**D**). *n* = 3, * *p* < 0.05, ** *p* < 0.01, *** *p* < 0.001 using two-way ANOVA. All graphs reported as mean ± SD.

In order to explore whether the influence of Smyd1 on IL-6 is exclusively mediated via NF-κB, EA.hy926 cells were transfected with vectors containing either the Smyd1 gene or without the specific gene insert in the presence/absence of the chemical NF-κB inhibitors PDTC and Bay-11-7082. Real-time RT-qPCR on lysates (Figure 4A,B) and ELISA analysis using supernatants (Figure 4C,D) revealed lower IL-6 expression (*p* < 0.001) and secretion (*p* < 0.01) in Smyd1-overexpressing cells after treatment of the cells with PDTC (real-time RT-qPCR: 39.5%; ELISA: 43.3%) or Bay 11-7082 (real-time RT-qPCR: 56.9%; ELISA: 33.6%) compared to the untreated Smyd1-overexpressing cells. Accordingly, lower IL-6 expression (*p* < 0.05) and secretion were found in EA.hy926 cells not subjected to Smyd1 transfection but treated with PDTC (real-time RT-qPCR: 45.5%; ELISA: 60.5%; Figure 4A,C) or Bay 11-7082 (real-time RT-qPCR: 46.4%; ELISA: 48.4%; Figure 4B,D).

### 3.5. The Methyltransferase Activity of Smyd1 Is Involved in the Regulation of IL-6 Expression

**Figure 5:**
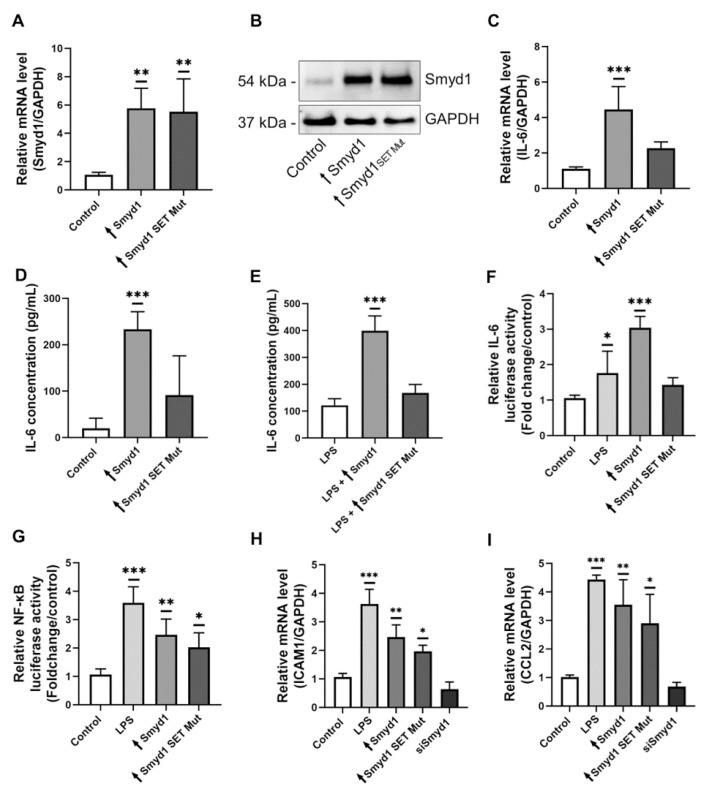
The methyltransferase activity of Smyd1 regulates IL-6 expression. (**A**,**B**) RT-qPCR for the quantification of Smyd1 mRNA levels (**A**) and immunoblotting for determination of Smyd1 protein levels (**B**) in total lysates of EA.hy926 cells transfected with the pCMV2-Smyd1-flag vector (↑Smyd1) or the Smyd1-SET-mutant vector (↑Smyd1 SET Mut). Expression values relative to control (transfection with vector lacking gene insert). *n* = 3, ** *p* < 0.01 using one-way ANOVA. Representative immunoblot of *n* = 3. (**C**) RT-qPCR for the quantification of IL-6 mRNA levels in EA.hy926 cells transfected with the pCMV2-Smyd1-flag vector (↑Smyd1) or the vector with a Smyd1 gene insert expressing a point mutation within the SET domain (↑Smyd1 SET mutant). Expression values relative to control (transfection with vector lacking gene insert). *n* = 5, *** *p* < 0.001 using one-way ANOVA. (**D**,**E**) ELISA for the determination of IL-6 concentrations in supernatants of non-stimulated (**D**) and 1 µg/mL LPS-stimulated (**E**) EA.hy926 cells transfected with a pCMV2-Smyd1-flag vector (↑Smyd1) or Smyd1-SET-mutant vector (↑Smyd1 SET Mut). *n* = 5, *** *p* < 0.001 using one-way ANOVA. (**F**) Relative luciferase activity of EA.hy926 cells transfected with a pBABE lucIL-6 reporter alone (Control), in combination with LPS stimulation (LPS) or co-transfected with the pCMV2-Smyd1-flag (↑Smyd1) or the vector with a mutated Smyd1 gene insert within the SET domain (↑Smyd1 SET Mut). Expression values relative to control. *n* = 3, * *p* < 0.05, *** *p* < 0.001 using one-way ANOVA. (**G**) Relative luciferase activity of EA.hy926 cells transfected with a NF-κB luciferase reporter gene alone (Control), in combination with LPS stimulation (LPS) or co-transfected with a pCMV2-Smyd1-flag (↑Smyd1) or a Smyd1-SET-mutant vector (↑Smyd1 SET Mut) vector. Expression values relative to control. *n* = 3, * *p* < 0.05, ** *p* < 0.01, *** *p* < 0.001 using one-way ANOVA. (**H**) RT-qPCR for the quantification of ICAM1 mRNA levels in EA.hy926 cells stimulated with LPS (1 µg/mL) for 24 h or transfected with Smyd1-specific siRNA, a pCMV2-Smyd1-flag vector (↑Smyd1) or the Smyd1-SET-mutant vector (↑Smyd1 SET mutant). Expression values relative to control. *n* = 3, * *p* < 0.05, ** *p* < 0.01, *** *p* < 0.001 using one-way ANOVA. (**I**) RT-qPCR for the quantification of CCL2 mRNA levels in EA.hy926 cells stimulated with LPS (1 µg/mL) for 24 h or transfected with Smyd1-specific siRNA, a pCMV2-Smyd1-flag vector (↑Smyd1) or the Smyd1-SET-mutant vector (↑Smyd1 SET mutant). Expression values relative to control (transfection with vector lacking gene insert). *n* = 3, * *p* < 0.05, ** *p* < 0.01, *** *p* < 0.001 using one-way ANOVA. All graphs reported as mean ± SD.

To investigate whether the methyltransferase activity of Smyd1 is involved in the regulation of IL-6 expression, EA.hy926 cells were transfected with a vector containing a gene insert that encodes a Smyd1 variant without methyltransferase activity due to a point mutation within the SET domain (Figure 5A,B and Figure S1J). In contrast to the active Smyd1-overexpressing EA.hy926 cells, this methyltransferase-inactive mutant had only a non-significant (*p* ≥ 0.05) impact on IL-6 expression, both at the mRNA level (Figure 5C) and the amount of secreted IL-6 (Figure 5D). Similar results were obtained for cells that were simultaneously Smyd1-transfected and LPS-stimulated (Figure 5E).

To characterize the role of Smyd1 in modulating IL-6 expression in more detail, EA.hy926 cells were transfected with an IL-6 promoter-driven luciferase reporter gene in combination with Smyd1 expression constructs (Figure 5F). LPS stimulation or transfection with the active Smyd1 gene resulted in higher luciferase activity (LPS stimulation: *p* < 0.05; +81%; Smyd1 transfection: *p* < 0.001, +202%), whereas overexpression of the Smyd1 mutant was not associated with higher luciferase activity rates (*p* ≥ 0.05, +35%). The effect of Smyd1 on NF-κB promoter activity was evaluated in transient transfection experiments with an NF-κB luciferase reporter construct (Figure 5G). If these EA.hy926 cells were exposed to 1 µg/mL LPS for 24 h, the promoter activity of NF-κB was strongly induced (*p* < 0.001; +247%; Figure 5G). Strikingly, co-transfection with the Smyd 1 vector or the Smyd1 mutant also increased the NF-κB promoter activity compared to the control transfected cells (Smyd1 transfection: *p* < 0.01, +130%; Smyd1 SET Mut transfection: *p* < 0.05, +88%; Figure 5G). 

In addition, the expression levels of established pro-inflammatory, NF-κB-dependent mediators were quantified in Ea.hy926 cells subjected to Smyd1 by RT-qPCR transfection. The experimental overexpression of Smyd1 and the methyltransferase-inactive mutant resulted in higher mRNA levels of ICAM1 (Smyd1 transfection: *p* < 0.01, +131%; Smyd1 SET Mut transfection: *p* < 0.05, +85%; Figure 5H), CCL2 (Smyd1 transfection: *p* < 0.01, +250%; Smyd1 SET Mut transfection: *p* < 0.05, +187%; Figure 5I) and VCAM1 (Smyd1 transfection: *p* < 0.01, +214%; Smyd1 SET Mut transfection: *p* < 0.05, +160%; data not shown). 

### 3.6. Smyd1 Affects the H3K4me3 Methylation Pattern of the IL-6 Promoter 

**Figure 6:**
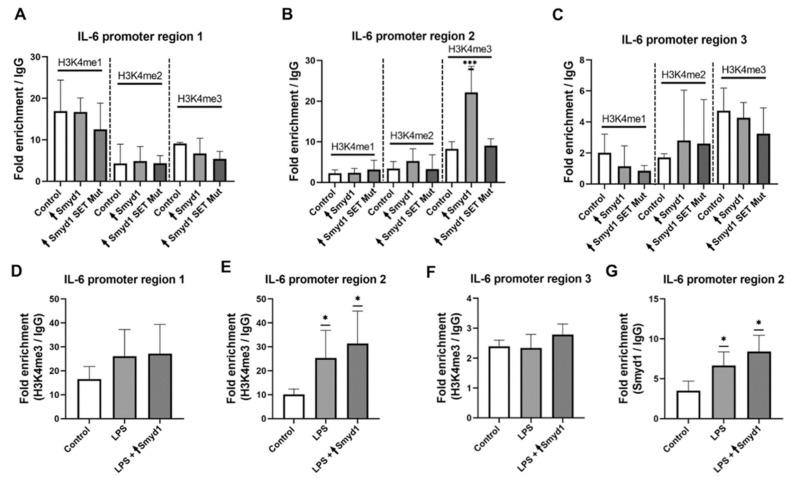
Smyd1 increases the H3K4me3 methylation status in the region 2 upstream of the transcription start site (TSS) within the IL-6 promoter. (**A**–**C**) ChIP assays were used to assess the degree of methylation of histone H3 atK4 (H3K4me1, H3K4me2 and H3K4me3) within the regions 1-3 of the IL-6 promoter in EA.hy926 cells that were transfected with either a pCMV2-Smyd1-flag vector (↑Smyd1) or a vector with a mutant Smyd1 gene lacking methylation activity due to a point mutation within the SET domain (↑Smyd1 SET mutant). An unrelated methylation-susceptible chromatin region with the GAPDH gene was included as a control in the ChIP-qPCR experiments. Three regions were selected: region 1 (−1469 to −1238; (**A**)), region 2 (−638 to −346; (**B**)) and region 3 (+150 to +347; (**C**)). The results were normalized to the negative (*IgG*) sample. *n* = 4. *** *p* < 0.001 using one-way ANOVA. (**D**–**F**) ChIP assays were used to assess the enrichment of the methylation site H3K4me3 within the IL-6 promoter in EA.hy926 cells that were transfected with a pCMV2-Smyd1-flag vector alone (↑Smyd1) or in combination with LPS (1 µg/mL) stimulation for 24 (LPS + ↑Smyd1). *n* = 3. * *p* < 0.05 using one-way ANOVA. (**G**), ChIP-qPCR for the Smyd1 level within the region 2 of the IL-6 promotor. *n* = 3. * *p* < 0.05 using one-way ANOVA. Data are shown as mean ± SD.

Since Smyd1 acts as an H3K4 histone methyltransferase, we next examined whether Smyd1 affects the methylation pattern of H3K4 within the IL-6 promoter region. Therefore, ChIP assays were used to evaluate the degree of H3K4 mono-, di- and trimethylation at three selected regions within the IL-6 promoter (IL-6 1, 2 or 3). EA.hy926 cells were transfected with an expression vector containing either the wild-type Smyd1 gene, a modified Smyd1 gene exhibiting a point mutation within the SET domain (extinguishing the histone methyltransferase activity) or without the specific gene insert as a negative control. Additional negative control experiments were performed using non-immune IgG. In addition, a representative active chromatin region (GAPDH) was included as a control in the ChIP RT-qPCR experiments (data not shown). Differences in the patterns of immunoprecipitated methylated proteins were not observed using the primers specific for region 1 (Figure 6A) within the distal region of the IL-6 promoter and region 3 (Figure 6C) downstream of the IL-6 downstream promoter element (DPE). In contrast, more H3K4 trimethylation events (*p* < 0.001, +572%; Figure 6B) were observed within the proximal IL-6 promoter region 2 (Figure 6B) in Smyd1-overexpressing cells compared with cells transfected with the Smyd1-SET mutant or the control vector. Co-stimulation of Smyd1-transfected EA.hy926 cells with LPS (1 µg/mL) for 24 h resulted likewise in an enrichment of H3K4 trimethylation within the proximal IL-6 promoter region 2 (LPS: *p* < 0.05, +150%; Figure 6E) but not in region 1 or region 3 (*p* ≥ 0.05; Figure 6D,F). In addition, region 2 showed weak binding of Smyd1, which was slightly increased after LPS stimulation and also after simultaneous transfection of Smyd1 (LPS: *p* < 0.05, +88%; Figure 6G). Only a very weak interaction of Smyd1 was found with region 1 (data not shown) and no interaction with region 3 (data not shown).

## 4. Discussion

This study aimed to investigate whether LPS mediates IL-6 up-regulation in ECs by involving the methyltransferase Smyd1. Major observations were as follows: (1) LPS stimulation of HUVECs and EA.hy926 cells resulted in higher Smyd1 mRNA and protein levels, (2) Smyd1 increased the expression of IL-6 in EA.hy926 cells and its secretion without a feedback effect of IL-6 on the Smyd1 expression, (3) Smyd1 influenced IL-6 expression partly via NF-κB and partly independently of NF-κB, and (4) the methyltransferase activity of Smyd1 led to more H3K4me3 methylation events within the IL-6 promoter.

The intrinsic Smyd1 expression at the mRNA and the protein levels was higher in HUVECs and EA.hy926 cells stimulated with LPS for 3 h and 24 h than in non-stimulated cells. These findings indicate that LPS induced Smyd1 up-regulation, which is, therefore, a downstream target of the LPS signaling cascade in ECs. Using a similar methodological approach, Smyd5 was previously identified to be part of the LPS signaling cascade in macrophages [13]. 

Interestingly, the Smyd1 expression level was higher after 3 h than after 24 h of LPS stimulation. A comparable time course in Smyd1 up-regulation was observed when ECs were incubated with the proinflammatory cytokine INF-γ for 3 h and 24 h [17]. We suggest that there is a molecular mechanism that terminates the induction of Smyd1 expression after a few hours, which is mediated by immunomodulatory molecules.

As recently published [17], Smyd1 is detected in both the nucleus and cytoplasm of ECs. Whereas the nuclear fraction of the Smyd1 pool probably exerts transcriptional control mainly through epigenetic regulation (histone methyltransferase activity/recruitment of HDACs), Smyd1-dependent methylation of non-histone targets in the cytoplasm may play a role in cell regulation [10]. Because Smyd1protein levels were increased in both cellular compartments in response to LPS stimulation, exposition of ECs to this bacterial fragment might lead to changes in transcriptional activity as well as cell signaling. 

RT-qPCR and immunoblot analyses revealed more IL-6 expression at the mRNA and the protein levels in Smyd1-transfected than in vector-transfected EA.hy926 cells. Furthermore, IL-6 was enriched in supernatants of EA.hy926 cells that were transfected with the vector encoding Smyd1 compared to those transfected with the vector lacking a gene insert. In contrast, the knockdown of Smyd1 expression caused less secretion of IL-6. Accordingly, the genetic loss of Smyd1 expression skeletal muscles resulted in a reduced expression of IL-6 in fetal mice prior to perinatal death [34]. Taken together, these findings imply that Smyd1 is an upstream regulator of IL-6 expression. Interestingly, IL-6 had no effect on Smyd1 expression, excluding a feedback interaction between these two proteins. 

ChIP analysis demonstrated more H3K4 trimethylation, commonly associated with the activation of transcription of nearby genes, within the region 2 of the IL-6 promoter in Smyd1-overexpressing EA.hy926 cells than in cells transfected with a mutant Smyd1 form exhibiting a point mutation within the SET domain (abolishing the histone methyltransferase activity) or in cells transfected with the vector lacking a specific gene insert. Strikingly, higher enrichment of H3K4 trimethylation within region 2 of the IL-6 promoter was detected in EA.hy926 cells stimulated with LPS. These data suggest that LPS triggers IL6-related immune responses, at least in part via up-regulation of Smyd1. A relationship between Smyd1 and the degree of methylation of H3K4 was previously demonstrated in heart and skeletal muscle cells [35,36,37,38,39]. Accordingly, SMYD1 elevates the levels of PGC-1α, a major regulator of mitochondrial biogenesis, in the adult heart of mice [39], and Isl1, a transcription factor important for embryogenesis, in the embryonic heart of mice [38] via H3K4me3 methylation in the corresponding promoter region. 

EA.hy926 cells overexpressing the Smyd1 form with mutated SET domain showed lower IL-6 mRNA and protein levels as well as lower IL-6 promoter activity when compared to active Smyd1-overexpressing EA.hy926 cells. These data also suggest that the methyltransferase activity of Smyd1 is involved in the increase in IL-6 expression in ECs, which was not clearly the case for other LPS-induced pro-inflammatory mediators tested in this study, such as ICAM1, VCAM1 and CCL2. 

The findings of the ChIP RT-qPCR assays suggest that Smyd1-formed H3K4me3 interacts with region 2 (but not with regions 1 and 3) of the IL-6 promoter to induce higher I-6 transcription rates. However, analysis using the UCSC Genome Browser revealed that regions 2 and 3 represent potential binding sites for nucleosomes trimethylated on histone-3. There are different possibilities to explain this contradiction. (1) The rate of H3K4 trimethylation is directly related to the local degree of methylation of the DNA [40]. For example, it was found that H3K4me3 is preferentially located at non-methylated CpG sites in CpG islands of promoters, which causes subsequent induction of the transcription of downstream genes. It is unclear how the UCSC Genome Browser takes into account that the DNA in the three regions can be dynamically methylated. (2) The H3K4me3/CpG island interaction involves other molecules, such as methyl-CpG-binding protein-2 [32] or Cfp1 [41]. If these proteins were located differently in the three DNA regions within the IL-6 promoter, it would be possible that more H3K4me3-modified nucleosomes are bound in region 2 and fewer in region 3 than predicted. (3) The stability of the H3K4me3 posttranslational modification depends on the activity of several histone deacetylases (HDACs; reviewed in 2019 [42]). If the concentration/activity of the HDACs was different between the three IL-6 regions, the stability and turnover of the H3K4 trimethylation label at the promoter would differ, which consequently could lead to variations in the IL-6 transcription rate. (4) Nucleosomes with H3K4me3 modification lead, depending on the location within the three regions of the IL-6 promoter, to the activation of different subsets of transcription factors and thus, possibly different IL-6 transcription rates. The promoter region of the human IL-6 gene is very complex [33]: it contains a TATA box in the core promoter region, several binding sites for transcription factors, such as NF-κB, SP (specificity protein)1, AP-1 (activator-protein-1), CREB (cyclic AMP-responsive element-binding protein), and C/EBP (CCAAT-enhancer-binding protein) in the proximal promoter region and binding sites for methylated histones and MeCPs in the distal promoter region. A summarizing list of transcription factors predicted by the UCSC Genome Browser is added as Table S1.

The transcription factor nuclear factor-kappa B (NF-κB) is a molecule that has an established impact on IL-6 expression and secretion in ECs [43]. To give an overview, stimulation with LPS leads to the activation of a receptor complex consisting of Toll-like receptor (TLR) 4, soluble CD14 and MD2 in ECs [44,45]. As a result, intracellular signaling pathways are up-regulated, leading to the activation of NF-κB in a complex process that is realized in two phases in most cells [44,45]. In an early activation process dependent on MyD88, LPS binding to the receptor complex induces the recruitment of several adaptor molecules (TIRAP, IRAK1 and IRAK4) that then activates the IKK complex, which finally leads to the proteolysis of members of the inhibitor of the NF-κB (IκB) family [44,46]. If these NF-κB inhibitors are absent due to their degradation, NF-κB is translocated to the nucleus, where it subsequently accumulates [47]. As a result, the transcriptional activity of NF-κB is enhanced to produce high mRNA levels of pro-inflammatory mediators, such as IL-6. After translation and secretion, these mediators can ultimately cause cell injury and sepsis [45]. A second pathway that leads to a late-phase, MyD88-independent activation of NF-κB in response to LPS binding, has not yet been observed in ECs [45]. It therefore remains to be clarified whether, and if so, at which step, Smyd1 converges with this NF-κB signal cascade.

To investigate whether Smyd1 influences IL-6 induction via NF-κB, we quantified the NF-κB levels in EA.hy926 cells transfected with Smyd1-expressing plasmids or Smyd1-specific siRNAs. Actually, both NF-κB expression and IL-6 transcriptional activity were higher in the Smyd1-overexpressing and lower in the Smyd1-downregulated (siRNA) cells when compared to the control vector-transfected cells. Furthermore, the nuclear accumulation and promoter activity of NF-κB were higher in the Smyd1-overexpressing ECs and lower in the Smyd1-downregulated ECs than in the control vector-transfected ECs. In contrast, IkBα levels were lower in the Smyd1-overexpressing ECs. These experimental data consistently suggest that Smyd1 is an upstream signaling molecule to increase the nuclear concentration and the transcriptional activity of NF-κB in ECs. However, because the promoter activity of NF-κB was also higher in cells transfected with the methyltransferase-inactive Smyd1 mutant, the effect on NF-κB appears to be independent of the methyltransferase activity of Smyd1.

Strikingly, the expression and secretion of IL-6 in EA.hy.926 cells incubated with two established chemical NF-κB blockers (PDTC and Bay 11-7082) was lower than in Smyd1-overexpressing ECs but still higher than in transfected control ECs. These findings imply that the effect of Smyd1 on IL-6 expression was not completely abolished by the chemical NF-κB inhibition and suggest that there is an additional molecular mechanism without NF-κB contribution by which Smyd1 affects IL-6 expression in ECs. In summary, our experimental findings allow the conclusion that Smyd1 up-regulates IL-6 transcription in ECs simultaneously by an NF-κB-dependent and an NF-κB-independent mechanism. 

In other cell systems, several molecular signaling pathways were identified through which IL-6 expression is induced without NF-κB involvement. (1) The transcription factor CCAAT-enhancer-binding protein delta (C/EBPδ) increased the IL-6 transcription in breast cancer cells [48] presumably by binding to one or both C/EBP binding sites within the IL-6 promoter region [49]. (2) The NF-κB-independent transcription factors CREB, AP-1 and C/EBP induced strong IL-6 mRNA up-regulation in mouse calvarial osteoblasts [50]. (3) The zinc-finger transcription factor Kruppel-like factor 4 (KLF4) has a dual function in the up-regulation of IL-6 in murine bone marrow-derived dendritic cells: in order to facilitate transcription, KLF4 binds to specific binding sites within the IL-6 promoter and also plays a role in chromatin remodeling at the IL-6 promoter region [51]. (4) Several examples demonstrated that the density of DNA methylation events within the IL-6 promoter contributes to the activation of IL-6 mRNA production. In rat hepatoma cells, loss of methylation events in the IL-6/STAT3 promoter was related to higher IL-6 levels and higher proliferation rates [52]. DNA hypomethylation was observed in the IL-6 promoter regions in synovial fibroblasts from osteoarthritis patients compared with those from non-diseased study participants [34]. The degree of DNA methylation influences the binding of methyl CpG binding proteins (MeCPs), which mediate the interaction with methylated histones [53]. (5) Histone modifications are also known to be involved in IL-6 gene expression [32,34,54,55]. In macrophages, epigenetic histone acetylation [54] and H3K36 di-methylation [55] regulate the transcription frequency of the IL-6 gene. Two mechanisms have been identified as to how these posttranslational modifications of histones alter transcriptional activity: (a) The chemical group attached to one histone can change the conformation of the complete nucleosome by directly increasing/reducing the accessibility of a DNA promoter for the binding of the RNA polymerase transcription complex. (b) Alternatively, an interaction of a modulating factor with the modified histone can indirectly influence transcription frequency, e.g., H3K4me3 was found to activate a large number of transcription factors, such as AP-1 [56], via interaction with the nucleosome remodeling factor (NURF) complex [57,58]. Considering the fact that the trimethylation of lysine 4 in histone 3 (H3K4me3) increased the IL-6 transcription rates in our study, we speculate that the histone methyltransferase is crucial for the mediation of the NF-κB-independent influence on the IL-6 expression in ECs. However, it is currently not possible to determine whether H3K4me3 directly or indirectly changes the IL-6 transcriptional activity in this cell system.

**Figure 7:**
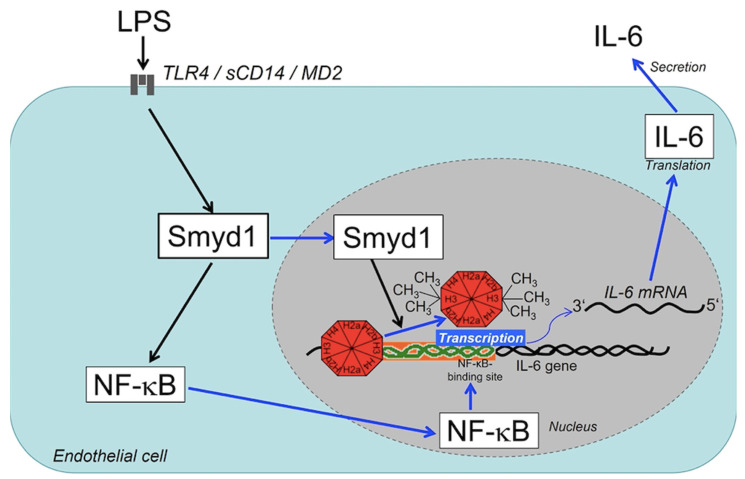
Schematic summary of the molecular model deduced from the experimental findings of this study. By binding to its specific receptor complex on the cell surface, LPS induces an up-regulation of Smyd1 in ECs. The corresponding intracellular increase of Smyd1 levels results in the activation of two signaling cascades. (1) More Smyd1 presence in the cytoplasm causes more frequent translocation of Smyd1 molecules into the cell nucleus. Enrichment of the methyltransferase Smyd1 in the nucleus results then in trimethylation of lysine 4 in histone 3 (H3K4me3) at region2 of the IL-6 promoter. This epigenetik modification leads to better accessibility of the promoter for the RNA polymerase transcription complex, so that the IL-6 transcription rate is increased. 2. More Smyd1 presence in the cytoplasm accompanied by IκBα down-regulation also up-regulates NF-κB expression leading to a larger cytoplasmic NF-κB pool. Subsequently, more NF-κB molecules diffuse into the nucleus, where NF-κB binds to a specific site within the promoter sequence (green-orange) of the IL-6 gene. As a consequence of the double activation of the promoter region, IL-6 transcription, as well as subsequent translation and secretion of the IL-6 protein, are accelerated resulting in higher IL-6 bioavailability. Black arrows indicate up-regulation, while blue arrows stand for intracellular transport.

In summary, our results reveal that Smyd1, as a previously unknown epigenetic modulator in human ECs, affects LPS-induced IL-6 expression and secretion in two ways: Firstly, through enhanced methylation of H3K4me3 and secondly through activation of the NF-κB signaling pathway (Figure 7). Since the IL-6 is crucial for the progression of acute inflammatory diseases, such as sepsis, this cytokine represents an important molecule within the LPS-signaling cascade. Our study, in addition to the emerging role in heart disease, suggests that Smyd1 may be of therapeutic interest. In addition to developing a specific Smyd1 inhibitor, future research should, therefore focus on uncovering additional target genes and identifying specific histone methylation sites of Smyd1.

## Figures and Tables

**Table 1:** Primer sequences, product sizes and annealing temperatures used to amplify the corresponding cDNA templates.

Template	Forward Primer	Reverse Primer	Product Size	Annealing Temperature
GAPDH	5′-ATG ACC TTG CCC ACA GCC TT-3′	5′-AAC TGC TTA GCA CCC CTG GC-3′	200 bp	60 °C
IL-6	5′-TGC CAG CCT GCT GAC GAA G-3′	5′-AGC TGC GCA GAA TGA GAT GAG-3′	90 bp	56 °C
Smyd1	5′-CTG GAG AAG CAG GAG CCA GTG TT-3′	5′-GCA TAG GCT TTG CAG ATC ATC CC-3′	257 bp	60 °C
ICAM1	5′-GGC CGG CCA GCT TAT ACA C-3′	5′-TAG ACA CTT GAG CTC GGG CA-3′	166 bp	58 °C
VCAM1	5′- TCA GAT TGG AGA CTC AGT CAT GT-3′	5′-ACT CCT CAC CTT CCC GCTC-3′	109 bp	62 °C
CCL2	5′-GAG AGG CTG AGA CTA ACC CAG A-3′	5′-ATC ACA GCT TCT TTG GGA CAC T -3′	259 bp	62 °C

**Table 2:** Sequences, PCR product sizes and position within the IL-6 gene of the primer pairs used in the ChIP-RT-qPCR assays. The information on the primer positions refers to the NCBI reference sequence for human interleukin 6: NG_011640.1.

Template	Forward Primer	Reverse Primer	Product Size	Position (for/rev)
IL-6 region 1	5′-TTT TCA CAC CAA AGA ATC CC-3′	5′-CTT ATT TAC CAA ACA TGG TGT-3′	231 bp	3532/3763
IL-6 region 2	5′-CAG GTG AAG AAA GTG GCA GA-3	5′-GAC CAG ATT AAC AGG CTA GAA-3′	292 bp	4363/4655
IL-6 region 3	5′-TCC TTA GCC CTG GAA CTG CC-3′	5′-AGG CAA CAC CAG GAG CAG CCC C-3′	197 bp	5150/5347

## Data Availability

All data generated or analyzed during this study are included in this published article.

## Supplementary Materials

The following are available online at https://www.mdpi.com/article/10.3390/cells10123515/s1, Figure S1: Densitometric quantification of tested protein expression levels on immunoblots, Supplementary Table S1: Compilation of the transcription factors that bind to the human IL-6 promoter sequences of the three DNA regions analyzed in the ChIP analysis, as calculated in the UCSC Genome Browser on Human Dec. 2013 (GRCh38/hg38).
