# Supplementary material for: The Methyltransferase Smyd1 Mediates LPS-Triggered Up-Regulation of IL-6 in Endothelial Cells
Source: Cells. 2021 Dec 13;10(12):3515. doi: 10.3390/cells10123515 (PMC8700543; doi:10.3390/cells10123515)
Supplement: Supplementary file 1 [file cells-10-03515-s001.zip › cells-1394038-supplementary.pdf]

Supplementary Table S1: Compilation of the transcription factors that bind to the human IL-6 promoter sequences of the three DNA regions analyzed in the ChIP analysis, as calculated in the UCSC Genome Browser on Human Dec. 2013 (GRCh38/hg38). Shown is also the position of the DNA sequence on chromosome 7 subjected to the bioinformatics analysis.

Region-1	Region-2	Region-3
chr7:22,725,678 - 22,725,910	chr7:22,726,509 - 22,726,802	chr7:22,727,296 - 22,727,494
IRF1<br>POUF<br>STAT1<br>TBR1/TBX2<br>SMAD3<br>FOS/JUN<br>ATF7<br>CREB1<br>NR2F1/ NR2F2<br>CUX<br>PRDM4	TCF<br>MYC<br>PROX1<br>ZNF341<br>OTX2<br>PITX3<br>GSC<br>ZNF460<br>POUF<br>ZNF136	ZNF281<br>ZNF148<br>KLF<br>GLI2<br>NFIC<br>ZIC1<br>MYF5<br>Ptf1a<br>BHLHA<br>NHLH<br>TFAP2<br>EBF

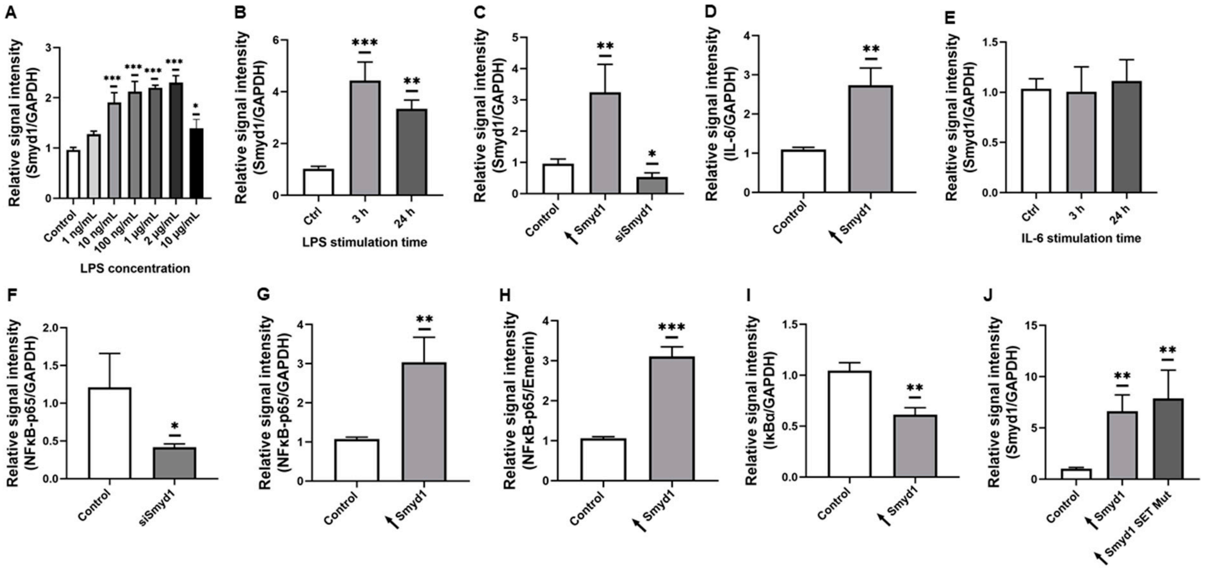

Supplementary Figure S1: Densitometric quantification of tested protein expression levels on immunoblots. (**A**) Immunoblotting for the determination of Smyd1 protein levels in total lysates of

EA.hy926 cells incubated with different concentrations of LPS ranging from 1 ng/mL to 10 µg/mL for 3 h in comparison to no LPS supplement (Control). $n = 3$. * $p < 0.05$, *** $p < 0.001$ using one-way ANOVA. (**B**) Immunoblotting for the determination of Smyd1 protein levels in total lysates of EA.hy926 cells after incubation with 1 µg/mL LPS for 3 h or 24 h in comparison to no LPS supplement (Ctrl). $n = 3$. ** $p < 0.01$, ***$p < 0.001$ using one-way ANOVA. (**C**) Immunoblotting for the determination of Smyd1 protein levels in pCMV2-Smyd1-flag vector (↑Smyd1) or Smyd1-specific siRNA transfected EA.hy926 cells. Expression values relative to control (transfection with vector lacking gene insert). $n = 3$, * $p < 0.05$, ** $p < 0.01$ using one-way ANOVA. (**D**) Immunoblotting for the determination of IL-6 protein levels in total lysates of EA.hy926 cells, which were transfected with either a pCMV2-Smyd1-flag vector (↑Smyd1) or a vector without specific gene insert (Control). Expression values relative to control. $n = 3$, ** $p < 0.01$ using Student *t*-test. (**E**) Immunoblotting for the determination of protein levels of Smyd1 in EA.hy926 cells that were stimulated with 10 ng/mL IL-6 for 3 h or 24 h. Expression values relative to control. $n = 3$. (**F**) Immunoblotting for the determination of NF-κB p65 levels in total lysates of EA.hy926 cells transfected with scrambled siRNA (Control) or Smyd1-specific siRNAs (siSmyd1). $n = 3$, * $p < 0.05$ using Student *t*-test. (**G**) Immunoblotting for the determination of NF-κB p65 levels in total lysates of EA.hy926 cells transfected with a vector lacking a specific gene insert (Control) or a pCMV2-Smyd1-flag vector (↑Smyd1) $n = 3$, ** $p < 0.01$ using Student *t*-test. (**H**) Immunoblotting for the determination of NF-κB p65 subunit levels in nuclear fractions of EA.hy926 cells that were transfected with a pCMV2-Smyd1-flag vector (↑Smyd1) or a vector without gene insert (Control) for 24 h. $n = 3$, *** $p < 0.001$ using Student *t*-test. (**I**) Immunoblotting for the determination of IκBα protein levels in total lysates of EA.hy926 cells transfected with a pCMV2-Smyd1-flag vector (↑Smyd1) or a vector without specific gene insert (Control). $n = 3$, ** $p < 0.01$ using Student *t*-test. (**J**) Immunoblotting for determination of Smyd1 protein levels in total lysates of EA.hy926 cells transfected with the pCMV2-Smyd1-flag vector (↑Smyd1) or the Smyd1-SET-mutant vector (↑Smyd1 SET Mut). Expression values relative to control (transfection with vector lacking gene insert). $n = 3$, ** $p < 0.01$ using one-way ANOVA. All graphs reported as mean ± SD.
